# Association of hypertension and depression with mortality: an exploratory study with interaction and mediation models

**DOI:** 10.1186/s12889-024-18548-0

**Published:** 2024-04-17

**Authors:** Huanhuan Huang, Fanchao Meng, Yanjie Qi, Xiuping Yan, Junhui Qi, Yuanzhen Wu, Yiwei Lin, Xu Chen, Fan He

**Affiliations:** 1 Beijing Key Laboratory of Mental Disorders, National Clinical Research Center for Mental Disorders & National Center for Mental Disorders, Beijing Anding Hospital, Capital Medical University, Beijing, 100088 China; 2 Advanced Innovation Center for Human Brain Protection, Capital Medical University, Beijing, China

**Keywords:** Hypertension, Depression, Mortality, Cardiovascular disease, Interaction, Mediation

## Abstract

**Background:**

The association of hypertension and depression with mortality has not been fully understood. We aimed to explore the possible independent or joint association of hypertension and depression with mortality. Their interaction effects on mortality and possible mediating role were also investigated.

**Methods:**

Associations of hypertension, depression, and their interaction with all-cause and cardiovascular disease (CVD) mortality were evaluated using multivariate Cox proportional hazards regression models. The mediation analysis was conducted with a Sobel test.

**Results:**

A total of 35152 participants were included in the final analysis. Hypertension and depression were independently associated with increased risk of all-cause and CVD mortality. The co-existence of hypertension and depression resulted in a 1.7-fold [95% confidence interval (CI): 1.3-2.1] increase in all-cause mortality and a 2.3-fold (95% CI: 1.4-3.7) increase in CVD mortality compared to those with neither of them. Hypertension and depression showed no significant multiplicative (P for interaction, 0.587) and additive interaction (P for relative excess risk of interaction, 0.243; P for Interaction on additive scale, 0.654) on all-cause mortality, as well as on CVD mortality. Depression did not mediate the relationship between hypertension and all-cause (Z=1.704, *P=*0.088) and CVD mortality (Z=1.547, *P=*0.122). Hypertension did not mediate the relationship between all-cause and CVD mortality as well.

**Conclusion:**

Hypertension and depression were related to all-cause and CVD mortality independently and the co-existence of them increased the risk of mortality. However, there is no interaction effect of them on mortality, and hypertension or depression did not mediate the association of each other with mortality.

## Introduction

Hypertension is common and affects more than 30% of the adult population worldwide. It is estimated that more than 1.28 billion people are suffering from hypertension around the world [1]. It has become one of the most critical and costly public health problems. High blood pressure is a major cause of disease burdens such as ischemic heart disease, stroke, other cardiovascular diseases (CVDs), and chronic heart disease [2]. More importantly, numerous studies have proved that hypertension remains a global risk factor for death, disability-adjusted life years, and life loss years [2–4].

Depression is a mood disorder that severely limits psychosocial functioning and diminishes the quality of life. Depression is also highly prevalent among the general population, and the lifetime prevalence of depression was reported to range from 2% to 21% worldwide [5]. Depression was projected to be the leading cause of disease burdens by 2023 due to its high prevalence (World Health Organization), causing substantial disability, diminished work productivity, and absenteeism. Abundant evidence showed that depression is associated with increased all-cause mortality including CVD mortality [6, 7].

Among the most important risk factors for mortality, the relationship between hypertension and depression has been studied extensively. The presence of these two symptoms together is not uncommon. Previous research has indicated that individuals with hypertension are more likely to suffer from depression [8]. At the same time, depression has been found to increase the risk of hypertension [9]. Evidence supports that the co-existence of depression and hypertension is related to higher functional disability [10]. In addition, comorbid depressive symptoms are reported to be associated with increased all-cause mortality in the population with self-reported hypertension [11].

While both hypertension and depression are associated with increased mortality, it is not clear whether the association between hypertension and mortality varies across different depression statuses or whether the association between depression and mortality differs among participants with or without hypertension. Given that hypertension and depression affect one another pathophysiologically as both hypertensive and depressive patients experience increased sympathetic tone and increased secretion of adrenocorticotropic hormone and cortisol [12], it is theoretically plausible to infer that there might be an interaction effect of them with mortality. Moreover, both of them might act as a mediator in the association of one another with mortality.

Herein, this study aimed to investigate the possible independent or joint association of hypertension and depression with all-cause and CVD mortality utilizing data from the National Health and Nutrition Examination Survey (NHANES) database. Their interaction effects on mortality and possible mediating role were also investigated.

## Methods

### Data source

The NHANES is a large, nationally representative cross-sectional survey conducted in the United States. It is a major project of the National Center for Health Statistics (NCHS), and it focuses on health conditions and behaviors, physical examination findings, and laboratory results. The survey was designed to sample the non-institutionalized population with a complex, stratified, multistage, probability cluster method. The study protocols were approved by the Ethics Review Board of the NCHS, and all participants provided written informed consent. The NHANES data are released in 2-year cycles. During each survey, participants undergo an interview in the household and then a clinical examination in the mobile examination center. We combined data from 2005-06 to 2017-18 as the NHANES used a different rating scale for depression before 2005. Detailed information about NHANES could be obtained from https://www.cdc.gov/nchs/nhanes/index.htm (accessed on September 13, 2023).

### Hypertension and depression assessment

Blood pressure was measured by trained staff, and mean values were determined as the mean of three or four readings according to the standardized protocol. Hypertension was defined as the systolic blood pressure of 130 mm Hg or greater, diastolic blood pressure of 80 mm Hg or greater, or the use of antihypertensive medications.

Depression was assessed by the Patient Health Questionnaire (PHQ-9) according to the Diagnostic and Statistical Manual of Mental Disorders, fourth edition. Participants were asked the frequency of depressive symptoms in the last two weeks. The total score of PHQ-9 was 0-27 points, of which 0-9 was no depression and 10-14, 15-19, and 20-27 was moderate, moderately severe, and severe depression, respectively. We used 10 points as the cutoff for clinically relevant depression as it has been proven to provide a favorable balance between sensitivity and specificity [13].

### Mortality assessment

Mortality information was obtained from the linked data from the Centers for Disease Control and Prevention [(https://www.cdc.gov/nchs/data-linkage/mortality-public.htm (accessed on September 21, 2023)]. The International Classification of Diseases-10 (ICD-10) was used to determine disease-specific mortality. CVD mortality was defined as ICD 100-109, I11, I13, I20-I51, and I60-I69. The follow-up ended when the people died or were censored through 31 December 2019.

### Covariates

Important covariates were identified in the literature on depression and hypertension. The covariates included age, gender, race/ethnicity, education, income, obesity, diabetes, dyslipidemia, history of CVD and cancer, physical activity, smoking, excessive alcohol use, and antidepressant use. Demographic or health-related information was obtained during the in-house interview. Blood samples were gathered at the mobile examination center and subsequently sent to central laboratories for the quantification of plasma glucose, hemoglobin A1c, and total cholesterol.

Race/ethnicity was self-reported and grouped into the following categories: Mexican American, non-Hispanic white, non-Hispanic black, and other. Educational level was determined by inquiring about the highest level of school attended or the highest degree obtained, and subsequently categorized as follows: less than high school, high school graduate, and college or higher. Income was quantified using the income-to-poverty ratio, a metric calculated by dividing the annual family income by the poverty threshold adjusted for family size and inflation. During the physical examination, weight and height were measured and the body mass index (BMI) was computed as the ratio of weight in kilograms to height in meters squared. Obesity was defined as a BMI of 30 or larger. Diabetes was defined as hemoglobin A1c ≥6.5%, fasting plasma glucose ≥126 mg/dL, or the use of antidiabetic medications. Dyslipidemia was defined as total cholesterol ≥240 mg/dL, or the use of lipid-lowering medications. History of CVD and cancer was ascertained by self-report at baseline, and CVD included congestive heart failure, angina, myocardial infarction, or stroke. Physical activity was assessed using the Global Physical Activity Questionnaire, and the overall physical activity level was computed as the sum of minutes engaged in moderate-intensity activities plus twice the minutes spent in vigorous-intensity activities [14]. Smoking status was determined through self-reported responses to questionnaires, inquiring whether a participant was currently smoking or not. Excessive alcohol use was defined as having an average intake of ≥5 drinks per day in the 12 months prior to the interview [15]. At the in-house interview, participants were asked about any prescription medications they had taken within the past 30 days. Antidepressant medications were identified using the second level of drug ingredient categorical codes. A combination of bupropion and naltrexone, primarily prescribed for addressing issues related to obesity, overweight, and weight-related medical concerns, was excluded [16].

### Statistical analysis

We limited our participants to non-pregnant adults aged 18 years or older. Those with missing information on hypertension, depression, or mortality were excluded. Statistical analysis was performed with R (R Development Core Team, Version 4.1.3). Primary sampling units, strata variables, and sampling weights were used in the analysis to account for the complexity of the survey design and to generate estimates representative of the noninstitutionalized US population. All statistical tests were 2-sided, and *P* < 0.05 was considered statistically significant [17]. We performed power analysis to evaluate the sample size required for models with time-to-event outcomes according to the literature [18]. The total person years of follow-up and number of participants should be at least 3315 person-years and 1529, respectively.

Continuous variables were expressed as mean (examined by student’s t-test) and category variables (examined by chi-square test) as percentages. Mortality was expressed as events per 1000 person-years of follow-up. Multivariate Cox proportional hazards regression models were used to examine the independent and joint association of hypertension and depression with all-cause and CVD mortality. The joint association of hypertension and depression with mortality was assessed by taking participants with neither hypertension nor depression as reference. Model 1 did not adjust any confounders. Model 2 was adjusted for age, gender, race/ethnicity, education, and income. Model 3 was adjusted for all the included covariates. In Model 3, we calculated the adjusted population-attributable fractions (PAFs), referring to the proportional risk of mortality that would have been avoided from hypertension or depression.

In interaction analyses, we evaluated multiplicative interaction with cross-product terms of hypertension with depression. We also evaluated additive interactions with relative excess risk owing to interaction (RERI) and the attributable proportion of interaction (AP) of total hazard owing to additive interaction. When the confidence interval (CI) of RERI and AP contained 0, there was no additive interaction.

We explored the mediating role of depression in the association between hypertension and all-cause and CVD mortality with a Sobel test [19]. We calculated β1 (representing the effect of hypertension on depression), β2 (representing the effect of depression on mortality when hypertension is not considered), and β3 (representing the direct effect of hypertension on mortality). The Sobel calculation [https://quantpsy.org/sobel/sobel (accessed on September 20, 2023)] provides the test statistics and *P*-values for evaluating the significance of mediating effects. The mediating role of hypertension in the association between depression and mortality was evaluated in a similar manner.

## Results

### Baseline characteristics of participants

A total of 67364 individuals finished the in-person interview and physical examination from 2005-06 to 2017-18. Among them, 40496 were adults aged 18 years or older. Then, 726 of them were excluded due to pregnancy and 4618 were excluded due to missing information on hypertension, depression, or mortality. Thus, a total of 35152 participants were included in the final analysis.

The baseline characteristics of participants stratified by hypertension or depression were presented in Table 1. The results showed that age, gender, race, education, obesity, diabetes, dyslipidemia, history of CVD and cancer, length of physical activity, smoking, and excessive alcohol use were statistically different among participants with and without hypertension. In addition, there were significant differences in gender, race, education, obesity, diabetes, dyslipidemia, history of CVD and cancer, length of physical activity, smoking, excessive alcohol use, and antidepressant use among participants with and without depression.

**Table 1:** Baseline characteristics of participants stratified by hypertension or depression status (NHANES 2005-2018)

Characteristics	Hypertension absent	Hypertension present	*P*-value	Depression absent	Depression present	*P*-value
Unweighted sample size	17740	17412		32059	3093	
Age, year	39.4 (0.2)	55.7 (0.3)	<0.001	46.9 (0.3)	46.4 (0.4)	0.306
Men, %	47.3 (0.4)	52.2 (0.5)	<0.001	50.6 (0.3)	36.9 (1.1)	<0.001
Non-Hispanic White, %	66.2 (1.3)	69.8 (1.3)	<0.001	68.2 (1.3)	63.6 (1.8)	<0.001
Less than high school, %	15.1 (0.6)	17.2 (0.6)	0.029	15.2 (0.5)	26.0 (1.1)	<0.001
IPR <1.3, %	20.9 (0.7)	18.5 (0.6)	0.506	18.3 (0.5)	38.1 (1.3)	<0.001
Obese, %	27.7 (0.6)	48.0 (0.6)	<0.001	36.1 (0.6)	47.4 (1.3)	<0.001
Diabetes, %	4.5 (0.2)	19.4 (0.4)	<0.001	10.8 (0.3)	17.7 (0.9)	<0.001
Dyslipidemia, %	17.0 (0.5)	42.2 (0.6)	<0.001	28.3 (0.4)	32.6 (1.1)	<0.001
History of CVD	3.6 (0.2)	14.6 (0.4)	<0.001	8.1 (0.2)	17.0 (0.9)	<0.001
History of cancer	6.8 (0.3)	14.5 (0.4)	<0.001	10.3 (0.3)	11.9 (0.9)	0.087
Length of PA, min/week	18.3 (0.4)	13.5 (0.3)	<0.001	16.4 (0.3)	13.4 (0.7)	<0.001
Current smoking, %	22.0 (0.6)	18.0 (0.4)	<0.001	18.6 (0.4)	39.1 (1.3)	<0.001
Excessive alcohol use, %	12.1 (0.4)	9.1 (0.4)	<0.001	10.5 (0.3)	13.4 (0.7)	<0.001
Antidepressant use, %	22.7 (0.8)	22.5 (0.6)	0.780	19.7 (0.5)	49.7 (1.6)	<0.001

All the data were presented as mean or percentage with standard error in parenthesis

*CVD* Cardiovascular disease, *IPR* Income-to-poverty ratio, *PA* Physical activity

### Independent and joint association of hypertension and depression with mortality

During a median follow-up of 7.4 years (interquartile range: 4.1-10.8 years), 3589 participants died and 1088 of them died of CVD. As shown in Table 2, hypertension was associated with increased odds of all-cause mortality in Model 1 [Hazard Ratio (HR): 3.9; 95%CI: 3.5-4.3], Model 2 (HR: 1.2; 95% CI: 1.1-1.3) and Model 3 (HR: 1.2; 95% CI: 1.0-1.3). It was associated with increased odds of CVD mortality in Model 1 (HR: 5.9; 95% CI: 4.8-7.3), Model 2 (HR: 1.5; 95% CI: 1.2-1.9), and Model 3 (HR: 1.5; 95% CI: 1.1-2.1). The fully adjusted PAFs analyses indicated 8.3% (95% CI: 1.9%-14.8%) of all-cause mortality and 21.7% (95% CI: 8.8%-34.6%) of CVD mortality would have been avoided if individuals did not suffer from hypertension. Similarly, depression was associated with an increased risk of all-cause mortality in Model 1(HR: 1.5; 95% CI: 1.3-1.7), Model 2 (HR: 1.8; 95% CI: 1.6-2.1), and Model 3 (HR: 1.4; 95% CI: 1.2-1.7). It was associated with an increased risk of CVD mortality in Model 1 (HR: 1.4; 95% CI: 1.1-1.7), Model 2 (HR: 1.9; 95% CI: 1.6-2.4), and Model 3 (HR: 1.5; 95% CI: 1.2-2.0). The fully adjusted PAFs analyses indicated 2.7% (95% CI: 1.4%-4.0%) of all-cause mortality and 3.1% (95% CI: 0.5%-5.7%) of CVD mortality would have been avoided if individuals did not suffer from depression.

**Table 2:** Independent association of hypertension and depression with all-cause and CVD mortality

	**Hypertension**		**Depression**	
**Number of participants**	**Absent**	**Present**	**Absent**	**Present**
	17740	17412	32059	3093
**All-cause mortality**				
Events/Person-years	796/140881	2793/124127	3192/242520	397/22489
Mortality rates^a^	4.5 (4.1-4.9)	17.2 (15.9-18.4)	9.7 (9.0-10.4)	15.1 (13.0-17.2)
Model 1	Reference	3.9 (3.5-4.3)	Reference	1.5 (1.3-1.7)
Model 2	Reference	1.2 (1.1-1.3)	Reference	1.8 (1.6-2.1)
Model 3	Reference	1.2 (1.0-1.3)	Reference	1.4 (1.2-1.7)
PAF (95% CI), %	8.3 (1.9-14.8)		2.7 (1.4-4.0)	
**CVD mortality**				
Events/Person-years	187/140881	901/124127	972/242520	116/22489
Mortality rates^a^	0.9 (0.7-1.1)	5.4 (4.8-6.0)	2.8 (2.5-3.1)	4.2 (3.2-5.1)
Model 1	Reference	5.9 (4.8-7.3)	Reference	1.4 (1.1-1.7)
Model 2	Reference	1.5 (1.2-1.9)	Reference	1.9 (1.6-2.4)
Model 3	Reference	1.5 (1.1-2.1)	Reference	1.5 (1.2-2.0)
PAF (95% CI), %	21.7 (8.8-34.6)		3.1 (0.5-5.7)	

Model 1 did not adjust any confounders. Model 2 was adjusted for age, gender, race/ethnicity, education, and income. Model 3 was adjusted for age, gender, race/ethnicity, education, income, obesity, diabetes, dyslipidemia, history of CVD, history of cancer, length of physical activity, smoking, alcohol use, antidepressant use, and substance use

*PAF* Population attributable fraction, *CVD* Cardiovascular disease, *CI* Confidence interval

^a^Per 1000-person years

In the joint analysis (Table 3), the co-existence of hypertension and depression resulted in a 5.4-fold (95% CI: 4.4-6.5), 2.1-fold (95% CI: 1.8-2.5), and 1.7-fold (95% CI: 1.3-2.1) increase in all-cause mortality compared to those without hypertension and depression in Model 1, Model 2, and Model 3, respectively. Similarly, the combination of hypertension and depression led to a 7.9-fold (95% CI: 5.5-11.4), 2.9-fold (95% CI: 2.0-4.2), and 2.3-fold (95% CI: 1.4-3.7) increase in CVD mortality compared to those without neither of them in Model 1, Model 2, and Model 3, respectively.

**Table 3:** Joint association of hypertension and depression with all-cause and CVD mortality

	**Hypertension absent, depression absent**	**Hypertension present, depression absent**	**Hypertension absent, depression present**	**Hypertension present, depression present**	**P for trend**
**Number of participants**	16315	15744	1425	1668	
**All-cause mortality**					
Events/Person-years	699/129845	2493/112675	97/11036	300/11453	
Mortality rates^a^	4.2 (3.8-4.7)	16.7 (15.4-18.0)	8.3 (6.0-10.6)	22.2 (18.5-25.8)	
Model 1	Reference	4.0 (3.6-4.5)	2.0 (1.5-2.7)	5.4 (4.4-6.5)	<0.001
Model 2	Reference	1.2 (1.1-1.3)	2.0 (1.5-2.7)	2.1 (1.8-2.5)	<0.001
Model 3	Reference	1.2 (1.0-1.4)	1.6 (1.1-2.3)	1.7 (1.3-2.1)	<0.001
**CVD mortality**					
Events/Person-years	164/129845	808/112675	23/11036	93/11453	
Mortality rates^a^	0.9 (0.7-1.0)	5.3 (4.7-5.9)	1.6 (0.8-2.4)	6.7 (4.8-8.7)	
Model 1	Reference	6.1 (5.0-7.5)	1.9 (1.1-3.2)	7.9 (5.5-11.4)	<0.001
Model 2	Reference	1.5 (1.2-1.9)	2.2 (1.3-3.6)	2.9 (2.0-4.2)	<0.001
Model 3	Reference	1.5 (1.1-2.1)	1.5 (0.8-3.0)	2.3 (1.4-3.7)	<0.001

Model 1 did not adjust any confounders. Model 2 was adjusted for age, gender, race/ethnicity, education, and income. Model 3 was adjusted for age, gender, race/ethnicity, education, income, obesity, diabetes, dyslipidemia, history of CVD, history of cancer, length of physical activity, smoking, alcohol use, antidepressant use, and substance use

*CVD* Cardiovascular disease, *CI* Confidence interval

^a^Per 1000-person years

### Interaction between hypertension and depression with mortality

Table 4 showed a positive multiplicative effect between hypertension and depression with all-cause mortality in Model 1 (P for interaction, 0.034). However, this multiplicative effect was insignificant in Model 2 and Model 3. An insignificant multiplicative effect was also observed between hypertension and depression with CVD mortality in Model 1, Model 2, and Model 3.

**Table 4:** Interaction between hypertension and depression with all-cause and CVD mortality

		All-cause Mortality	CVD mortality
Interaction on multiplicative scale, HR (95% CI)	Model 1	0.7 (0.5-1.0), *P=*0.034	0.7 (0.4-1.2), *P=*0.208
	Model 2	0.8 (0.6-1.2), *P=*0.426	0.9 (0.5-1.6), *P=*0.659
	Model 3	0.9 (0.6-1.4), *P=*0.587	1.0 (0.5-2.1), *P=*0.955
Interaction on additive scale, RERI (95% CI)	Model 1	0.4 (-0.5-1.3), *P=*0.412	0.9 (-3.9-5.6), *P=*0.723
	Model 2	-0.1 (-0.6-0.4), *P=*0.605	0.2 (-1.1 -1.5), *P=*0.758
	Model 3	-0.1 (-0.6-0.4), *P=*0.243	0.2 (-0.9-1.3), *P=*0.668
Interaction on additive scale, AP (95% CI)	Model 1	0.1 (-0.1-0.2), *P=*0.444	0.1 (-0.5-0.7), *P=*0.704
	Model 2	-0.1 (-0.3-0.2), *P=*0.586	0.1 (-0.4-0.5), *P=*0.753
	Model 3	-0.1 (-0.5-0.4), *P=*0.654	0.1 (-0.4-0.6), *P=*0.665

Model 1 did not adjust any confounders. Model 2 was adjusted for age, gender, race/ethnicity, education, and income. Model 3 was adjusted for age, gender, race/ethnicity, education, income, obesity, diabetes, dyslipidemia, history of CVD, history of cancer, length of physical activity, smoking, alcohol use, antidepressant use, and substance use

*HR* Hazard ratio, *RERI* Relative excessive risk due to interaction, *AP* Proportion attributable to the interaction, *CVD* Cardiovascular disease

In addition, hypertension and depression showed an insignificant additive interaction on all-cause mortality in Model 1, Model 2, and Model 3. Similarly, this additive interaction was also insignificant between hypertension and depression with CVD mortality in Model 1, Model 2, and Model 3.

### Mediation analysis

**Fig. 1:**
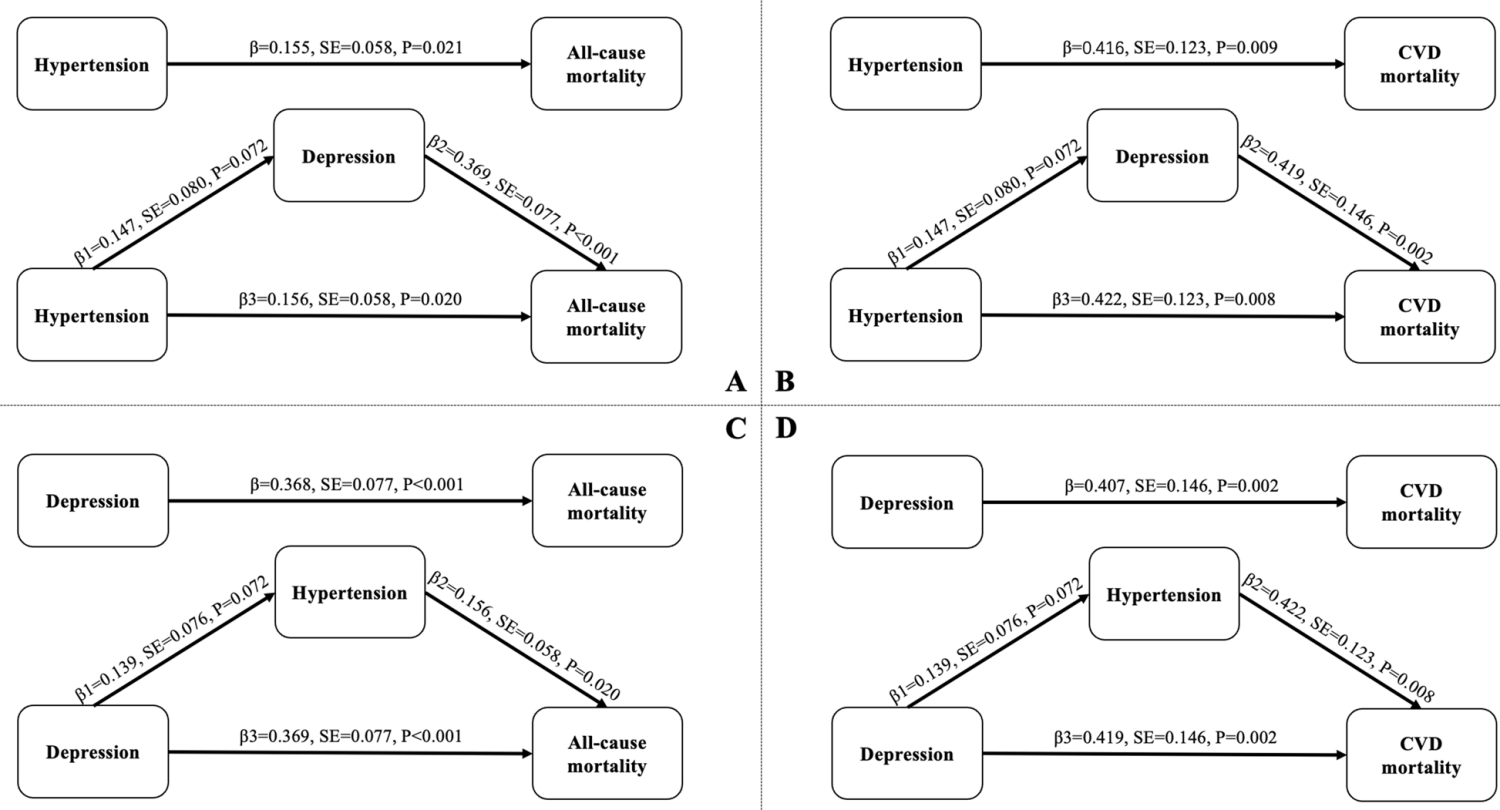
Schematic diagram of mediation analysis by Sobel Test. β represents the total effect of hypertension or depression on all-cause or cardiovascular mortality. β1 represents the effect of hypertension on depression or the effect of depression on hypertension. β2 represents the effect of depression on mortality when hypertension is not considered, or the effect of hypertension on mortality when depression is not considered. β3 represents the direct effect of hypertension or depression on mortality

As shown in Fig. 1, after adjusting for potential covariates, hypertension was significantly associated with all-cause mortality (β=0.155, SE=0.058, *P=*0.021). Next, when the mediator, depression, was entered into the regression model, hypertension remained a significant predictor of all-cause mortality (β3=0.156, SE=0.058, *P=*0.020). On the other hand, the mediator, depression, emerged as a significant predictor of all-cause mortality (β2=0.369, SE=0.077, *P*<0.001). The association between hypertension and depression was insignificant after adjusting for covariates (β1=0.147, SE=0.080, *P=*0.072). The results of the mediation analysis showed that depression did not mediate the relationship between hypertension and all-cause mortality (Z=1.704, *P=*0.088). Similarly, depression did not mediate the relationship between hypertension and CVD mortality (Z=1.547, *P=*0.122) (Fig. 1).

We also examined whether hypertension was a potential mediator between the association of hypertension and all-cause and CVD mortality. The results of mediation analysis were insignificant for all-cause mortality (Z=1.512, *P=*0.130) (Fig. 1) and CVD mortality (Z=1.614, *P=*0.107) (Fig. 1).

### Sensitivity analysis

We performed several sensitivity analyses to verify the robustness of the results. First, we conducted a sensitivity analysis by gender (male and female) and age (< 45 years and >= 45 years). Second, we excluded participants who reported a history of CVD, as they were more predisposed to CVD mortality. Third, we excluded participants who reported substance use in the last year. Fourth, we defined hypertension as a systolic blood pressure of 140 mmHg or diastolic blood pressure of 90 mmHg, according to the World Health Organization. Lastly, we excluded participants who died within the first two years of follow-up. The results of the sensitivity analyses remained similar to those of the main analyses.

## Discussion

In this large sample cross-sectional study, we examined the possible association of hypertension and depression with mortality with different statistical methods. Our study proved that both hypertension and depression are independently associated with increased odds of all-cause and CVD mortality. In addition, the joint analysis confirmed the co-occurrence of hypertension and depression had a cumulative effect and put individuals at a higher risk for mortality. This result was consistent with previous findings [11, 20]. For example, using a nationally representative longitudinal survey focusing on elderly aged 60 or above, Kuo et al., proved that the group of participants with both hypertension and depression showed 1.54 (95% CI: 1.29-1.83) times increased risk of mortality compared to the group with neither hypertension nor depression [20]. In addition, we calculated the PAFs for hypertension and depression. It seems like hypertension contributed to a larger proportion of mortality (8.3% versus 2.7%), especially CVD-related mortality (21.7% versus 3.1%), than depression. These results together suggested that people with both hypertension and depression should be more rigorously monitored to improve their outcomes. The hypertension-related health condition is worth closer attention.

We examined the interaction of hypertension and depression with mortality, which, according to our knowledge, has not been explored by previous research. Before the study, we expected there might be a significant interaction effect as hypertension increased the risk of depression through physical or biological changes [21] and depression had adverse effects on cardiovascular health in clinical and sub-clinical aspects [22, 23]. This two-way spiral relationship could be explained by several mechanisms. From the epidemiological aspects, there was an increased prevalence of hypertension in depressed patients [24] and an increased prevalence of depression in hypertensive patients as well [25]. From the pathophysiological aspects, the underlying relationship between hypertension and depression probably involves a shared genetic vulnerability, the sympathetic nervous system, and stress hormones [12]. For example, a study demonstrated that subjects with a family history of hypertension displayed higher levels of blood pressure and heart rate during mental stress tasks and recovery. They also reported more depression during tasks, implying the involvement of genetic factors in cardiovascular hyperactivity [26]. A large amount of evidence showed that sympathetic hyperactivity is a specific feature of hypertension [27, 28]. There is also reported evidence showing depression was linked to the altered functioning of the autonomic nervous system, especially to increased sympathetic activity and/or decreased parasympathetic activity [29, 30]. From the clinical aspect, the presence of depression can impair the management and prognosis of hypertension. Depressed patients might cooperate less with treatment regimens due to cognitive limitations or a withdrawn coping style. The use of antihypertensive medications can potentially induce depressive symptoms, which can complicate the assessment of a causal relationship between depression and medication adherence. Conversely, the side effects of antidepressants would lead to the presence of hypertension in depressive patients. Tricyclic antidepressants and monoamine oxidase inhibitors have been associated with changes in systolic and diastolic blood pressure [31]. A Meta-analysis demonstrated a dose-dependent increase in blood pressure after venlafaxine treatment [32].

There is a lot of enthusiasm for studying interactions as it can elucidate the biological mechanisms underlying disease etiology and provide insights into the benefits of behavioral modifications to one risk factor within subgroups of individuals defined by another risk factor [33].

However, our study did not show a multiplicative or additive interaction effect of hypertension and depression on the risk of all-cause and CVD mortality after adjusting for the potential covariates. This result suggested that the effect of hypertension on mortality remained unchanged across the participants with or without depression and vice versa. The underlying reason has not been explained. Perhaps there is a certain degree of association between hypertension and depression that would increase the prevalence of hypertension among depressive individuals or the prevalence of depression among hypertensive individuals. However, this association was not strong enough to change the final health outcomes.

We also performed a mediation analysis to explore if hypertension or depression mediated the relationship of each other with mortality. The results of mediation analysis were insignificant as well, which suggested that the treatment of depression would not affect the mortality related to hypertension or that the modification of hypertension would not lead to a change in the mortality related to depression. This result was not surprising as the interaction analysis had indicated a weak association between hypertension and depression that might not change the health outcomes.

This study has strengths in its large sample size as well as the reliability of the NHANES data. The baseline information of the participants was cross-sectionally collected and their outcomes were obtained after a relatively long period of follow-up. However, there are also some limitations. First, this study was observational, and it is unlikely to determine the causal effect of hypertension or depression on the risk of mortality. Prospective studies are needed to further verify our results. Second, the NHANES used self-reported questionnaires rather than clinical interviews for the diagnosis of depression, making it susceptible to recall and information bias. However, PHQ-9 was a well-validated and accepted tool for use in screening large populations for depression. Thirdly, there was a potential overlap between depression and other psychiatric disorders, such as anxiety. However, information on these other disorders was not available in the NHANES, and thus we were unable to include them as covariates in the models. Lastly, CVD mortality in the NHANES was ascertained using linkage data, which might introduce some uncertainty.

## Conclusion

To our knowledge, this was the first study to explore the association of hypertension and depression on mortality. This study suggested that hypertension and depression were related to all-cause and CVD mortality independently and the co-existence of them increased the risk of mortality. However, there is no interaction effect of hypertension and depression on mortality, and hypertension or depression did not mediate the association of each other with mortality.

## Data Availability

No datasets were generated or analysed during the current study.

## Abbreviations

AP: Attributable proportion of interaction
BMI: Body mass index
CI: Confidence interval
CVD: Cardiovascular disease
HR: Hazard ratio
NCHS: National Center for Health Statistics
NHANES: National Health and Nutrition Examination Survey
PAFs: Population-attributable fractions
PHQ-9: Patient Health Questionnaire
RERI: Relative excess risk owing to interaction
